# Quantifying Drug Wastage and Economic Loss of Chemotherapy Drugs at an Adult Oncology Care of a Tertiary Care Public Hospital in India

**DOI:** 10.7759/cureus.49242

**Published:** 2023-11-22

**Authors:** Rutuja Fulsoundar, Neha Kadhe, Swati Patil, Shweta Ghate, Sudhir Pawar

**Affiliations:** 1 Pharmacology and Therapeutics, Lokmanya Tilak Municipal Medical College & Hospital, Mumbai, IND

**Keywords:** drug wastage, cost-effectiveness, single dose vials, sharing and rounding of doses, health economics

## Abstract

Background and objective

New drugs have revolutionized cancer care, but their high cost requires cost-effectiveness studies. However, these studies only consider optimal use, neglecting real-world wastage. We aim to assess chemotherapy drug wastage and financial loss in our adult oncology care.

Methods

A total of 100 adult patients attending daycare oncology were prospectively evaluated. The total dose of parenteral anticancer drug, the amount administered, and the amount of drug wasted were recorded for each patient. The economic loss estimation was done considering the unit cost for the drug.

Results

Our study evaluated 157 parenteral drug administrations of 10 different anticancer drugs in 100 enrolled patients. The most common diagnosis was breast cancer (39/100; 39%), and the most commonly prescribed drugs were paclitaxel (36/157; 23%) and cyclophosphamide (21/157; 13%). However, the wastage percentage varied from 6% to 35.06%, and the overall wastage estimated was 16,298 mg (20.06%) of the total drug procured. Notably, the highest proportion of drug wastage was observed for carboplatin (2,525/7200 mg; 35.06%), whereas oxaliplatin, gemcitabine, 5-FU, and cisplatin wastage were more than 20% of the ordered drug. The total cost of the chemotherapy drug procured was 7,26,005 INR (8,738.78 USD), and drug wastage amounted to 17.14% of the total drug cost, resulting in an economic loss of 1,24,485 INR (1,498.40 USD). Gemcitabine (542.86 USD), oxaliplatin (452.66 USD), and paclitaxel (286.15 USD) were responsible for the maximum cost of wastage.

Conclusion

Drug wastage and financial loss are significant for carboplatin, oxaliplatin, and gemcitabine, with small proportions of paclitaxel also contributing to economic loss. Possible solutions include planning pharmacy inventory for multiple vial sizes and drug-wise batching strategies to facilitate vial sharing. However, these approaches may present challenges. The pharmaceutical industry can consider initiatives such as providing varying packaging sizes to minimize drug wastage.

## Introduction

In 2020, the National Cancer Registry Programme (NCRP) of India estimated that there were 1.39 million new cases of cancer [1]. This highlights the significant burden of cancer in India and underscores the importance of research into improving cancer care and treatment. It is worth noting that, as per reports, several types of cancer are predominant among men, including cancers of the lung, mouth, stomach, and esophagus, whereas breast and cervical cancer are more common among women. Chemotherapy is a widely used treatment modality for various kinds of cancer, which can be administered either through a single drug or a combination of drugs. However, it is imperative to recognize that most chemotherapeutic agents have a narrow therapeutic index and a steep dose-response relationship. Even minute variations in the administered dose can lead to severe and life-threatening toxicity in some individuals or under-dosing in others, which can potentially compromise cancer outcomes. Moreover, chemotherapy drugs are prescribed based on the patient's body surface area (BSA), which can vary significantly and may not necessarily align with the available vial sizes, leading to drug wastage [2]. Drug wastage is defined as the amount of drug dispensed but discarded without administering it to a patient. It is the difference between the drug amount in a vial (especially a single dose vial) and the administered amount [3].

In India, one of the challenges faced by cancer patients and their caregivers is out-of-pocket expenditure on chemotherapy agents, which accounts for more than 75% of the cost [4]. Consequently, the healthcare system experiences a substantial financial setback. One of the primary solutions recommended to address the mounting global issue of escalating costs associated with cancer medications is to reduce the price of anticancer drugs [5].

Given the circumstances, it becomes even more crucial to assess the extent of such wastage and its associated costs so that a robust preventive strategy can be devised. Therefore, the purpose of this study was to estimate drug wastage and its economic impact concerning parenteral chemotherapy drug use in the adult oncology unit of our tertiary care hospital.

## Materials and methods

A single-center, cross-sectional, observational study was undertaken at an adult oncology day care unit of a tertiary care hospital in India spanning from December 2019 to May 2021. Before the commencement of the study, ethical approval was obtained from the Institutional Ethics Committee (IEC) (IEC No: IEC/382/19), and written informed consent was obtained from all the participants before their enrollment. The sample size for this study included 100 adult patients attending the oncology daycare unit during the study period. Patients who had their chemotherapy details recorded previously were excluded from the study.

The recorded chemotherapy details included the drug name, dose in mg/kg or mg/m2, total calculated dose, formulation details (strength [mg], unit size, single dose or multiple dose vial), and the actual drug consumed (mg) from the procured vial. Any remaining drug that was discarded was recorded as a wasted drug. The cost of the drug was estimated in Indian rupee (INR) and converted to US dollars (USD) (average exchange rate of 1 USD in 2023 was 83.07 INR). Drugs were obtained from the purchase order from the hospital pharmacy for the proportion of the discarded drug.

Additionally, we assessed whether the prescribed chemotherapy doses matched the vial size and strength availability at our institution and in the Indian market, as per the Current Index of Medical Specialities (CIMS) 2022. We also estimated the instances where drug administrations matched the vial strength available at our institution. We also noted any measures taken to minimize drug wastage, such as rounding off of the dose or sharing of a vial, if any.

Statistical analysis

All the data collected were entered into Microsoft Excel 2019 Version (Microsoft Corp., Redmond, WA). Descriptive statistics was employed for assessing the outcome measures. Continuous variables were expressed as mean ± standard deviation (SD), whereas categorical variables were expressed as percentages and frequencies.

## Results

During the period of our study, a total of 100 patients undergoing treatment at an oncology day care unit received 157 parenteral drug administrations of 10 different oncological drugs. The mean age of the patients was 49 (±10.74) years, with a predominance of female patients (male/female= 27/73), and the mean BSA was 1.47. Among 15 different carcinomas, breast cancer was the most prevalent, accounting for 39 of cases, followed by ovarian cancer (12 cases), and pancreatic carcinoma (10 cases). A detailed distribution of the types of cancer is presented in Table 1.

**Table 1:** Type of cancer (n=100)

System-wise distribution	Cancer	n
Reproductive system	Breast	39
	Ovary	12
	Cervix	2
	Vulva	1
	Prostate	1
Gastrointestinal tract	Pancreas	10
	Gall bladder	9
	Colon	6
	Stomach	6
	Esophagus	4
	Rectum	4
	Bladder	1
Oral cavity	Tongue	2
Respiratory tract	Lung	2
Other	Squamous cell carcinoma	1

Among the 157 parenteral chemotherapy drug administrations of 10 different drugs, paclitaxel (23%) was the most frequently prescribed drug, followed by cyclophosphamide (13%), whereas doxorubicin and oxaliplatin accounted for around 12% each, and gemcitabine constituted approximately 11%. The percentage of each of the different drugs used is presented in Table 2.

**Table 2:** Frequency of chemotherapy drug observed (n=157) 5-FU, fluorouracil

Drugs	Total number of drug administrations (n)	Percentage (%)
Paclitaxel	36	23
Cyclophosphamide	21	13
Doxorubicin	19	12
Oxaliplatin	19	12
Gemcitabine	18	11
Carboplatin	15	9.6
Cisplatin	14	8.9
5-FU	10	6.4
Docetaxel	3	1.9
Irinotecan	2	1.3

Drug wastage

In our study, the wastage percentage for individual drugs varied from 6% to 35.01%, and the overall wastage was estimated to be 16,298 mg out of 81,240 mg, i.e., 20.06% of the total drug procured (Table 3). Notably, the highest proportion of drug wastage was observed for carboplatin (35.06%), which was prescribed for various carcinomas such as ovarian, esophageal, breast, and lung, followed by oxaliplatin (27.09%) and gemcitabine (25.62%). For drugs with higher consumption rates, such as carboplatin, oxaliplatin, gemcitabine, 5-FU, and cisplatin, the wastage percentage was more than 20% of the ordered drug.

**Table 3:** Drug wastage for individual drugs (in mg) (n=157) 5-FU, fluorouracil

Drug	Total number of drug administrations (n)	Total amount of available drug in vials (mg)	Total amount of drug consumed (mg)	Total amount of drug wasted (mg)	Percentage of drug wastage (95% CI)
Carboplatin	15	7,200	4,675	2,525	35.06 (16.29-42.50)
Oxaliplatin	19	3,100	2,360	840	27.09 (14.18-31.08)
Gemcitabine	18	32,000	23,800	8,200	25.62 (15.42-30.13)
5-FU	10	7,500	5,880	1,620	21.6 (5.29-27.10)
Cisplatin	14	1,100	848	252	22.90 (6.53-27.32)
Docetaxel	3	420	370	50	11.9 (4.30-25.13)
Cyclophosphamide	21	21,000	19,020	1,980	9.42 (7.83-19.59)
Paclitaxel	36	6,560	5,924	636	9.69 (5.96-12.47)
Doxorubicin	19	1,860	1,695	165	8.87 (5.18-12.18)
Irinotecan	2	500	470	30	6 (4.79-14.79)

Cost of drug wastage

The overall drug wastage in the 157 drug administrations studied was found to be 20.06%. The total cost of the chemotherapy drugs procured was estimated to be 7,26,005 INR (8,738.78 USD), whereas the total drug wastage amounted to 17.14% of the total drug cost, resulting in an economic loss of 1,24,485 INR (1,498.40 USD). As shown in Table 4, gemcitabine (45,100 INR/542.86 USD), oxaliplatin (37,606 INR/452.66 USD), and paclitaxel (23,773 INR/286.15 USD) were responsible for the maximum cost of wastage.

**Table 4:** Details of cost of drug wastage (INR) (n=157) 5-FU, fluorouracil

Drug	Total number of drug administrations (n)	Total cost of drug (INR)	Total cost of wasted drug INR (USD)	Percentage cost of drug wasted (95% CI)
Carboplatin	15	30,600	8,961 (107.86)	29.28 (16.61-42.96)
Oxaliplatin	19	1,44,309	37,606 (452.66)	26.05 (14.18-31.07)
Gemcitabine	18	1,92,500	45,100 (542.86)	23.42 (15.42-30.13)
5-FU	10	352	90 (1.08)	25.56 (8.60-40.93)
Cisplatin	14	6,584	1,571 (18.91)	23.86 (6.40-33.11)
Docetaxel	3	45,533	3,338 (40.18)	7.33 (6.03-24.78)
Cyclophosphamide	21	2,100	243 (2.92)	11.57 (6.84-16.29)
Paclitaxel	36	2,56,198	23,773 (286.15)	9.27 (6.45-13.58)
Doxorubicin	19	31,829	2,843 (34.22)	8.93 (5.30-12.36)
Irinotecan	2	16,000	960 (11.56)	6 (4.79-14.79)

Chemotherapy drug dose and vial size matching

The frequency of vial strengths exactly matching the prescribed dose was found to be the highest for paclitaxel (n=6), cisplatin (n=5), doxorubicin (n=5), and oxaliplatin (n=3). However, for carboplatin, gemcitabine, and fluorouracil (5-FU), not a single vial strength matched the prescribed dose. The frequency of available vial strengths that exactly matched the prescribed dose for each drug is presented in Table 5.

**Table 5:** Details of available vial strengths and matching with prescribed dose (n=157) *NA, not applicable (as all patients received varying doses) 5-FU, fluorouracil

Drug	Total number of drug administrations (n)	Commonest dose prescribed (mg)	Median dose prescribed (mg, range)	Frequency of administrations with vials matching the prescribed dose (n)	Formulations available in our setup (mg)	Formulations available in the Indian market (mg)
Carboplatin	15	300	300 (100-700)	0	450	150, 450
Oxaliplatin	19	120	120 (100-180)	3	50, 100, 150	50, 100, 150
Gemcitabine	18	1,200	1,375 (1,200-1,800)	0	1,000	200, 1,000, 1,400
5-FU	10	730	730 (480-780)	0	500	250, 500
Cisplatin	14	50	55 (40-135)	5	50	10, 50
Docetaxel	3	NA	116 (100-150)	1	80	80, 120
Cyclophosphamide	21	900	900 (750-1,100)	2	1,000	200, 1,000
Paclitaxel	36	140	140 (75-302)	6	100, 260, 300	30, 100, 260, 300
Doxorubicin	19	90	90 (60-110)	5	50, 10	50, 10
Irinotecan	2	NA*	235 (200-270)	1	100	40, 100

Measures undertaken to curb wastage

The measures to minimize drug wastage, such as sharing or rounding of doses, were employed in only 23 out of 157 drug administrations, which accounts for 14.6%. The drugs in which wastage was mitigated due to the sharing and rounding of doses are listed in Table 6.

**Table 6:** Measures used to curb wastage (n=23) 5-FU, fluorouracil

Drugs	Sharing of vials (n)	Rounding of dose (n)
5-FU	1	3
Carboplatin	2	0
Cyclophosphamide	0	1
Gemcitabine	4	3
Oxaliplatin	3	1
Paclitaxel	0	5
Total	10	13

## Discussion

The cost of anti-cancer drugs is rising, and, as a result, healthcare providers are increasingly assessing the cost-effectiveness of these treatments [6]. This helps to determine whether the benefits of a particular drug outweigh its cost and enables providers to allocate their resources more effectively. Ultimately, it can lead to a reduction in the financial burden of cancer treatment for patients and healthcare systems.

However, these manufacturer-sponsored cost-effectiveness analyses routinely fail to account for the cost of drug wastage giving an inaccurate estimate. Our study revealed that 20.06% of the dispensed drug was wasted, resulting in an economic loss of INR 1,24,485 (1,498.40 USD), which accounts for 17.14% of the total procurement cost. This finding is consistent with a drug wastage study conducted in India by Gopisankar et al., where 19.72% of drug wastage accounted for a financial loss of 17.14% of the drug procurement cost [7]. However, in contrast, a study by D'Souza et al. observed that 6.06% of drug waste accounted for 11.11% of the total financial loss [8]. The disparity in these findings could be attributed to differences in the drug use spectrum, vial sizes available for use, and the mitigation strategies that were in place during the studies.

Our study indicated that the highest percentage of drug wastage was observed for carboplatin (35.06%; 95% CI: 16.29-42.50%), which was primarily used for the treatment of ovarian, esophageal, breast, and lung carcinomas. This was followed by oxaliplatin (27.09%; 95% CI: 14.18-31.08%), which was used for the treatment of stomach, colon, rectal, pancreatic, and ovarian carcinomas. Gemcitabine (25.65%; 95% CI, 15.42-30.13%) was the third most commonly wasted drug, which was used for the treatment of ovarian, pancreatic, and gallbladder carcinomas. Additionally, other drugs, such as cisplatin and 5-FU, showed drug wastage percentages of 22.90% (95% CI: 6.53-27.32%) and 21.6% (95% CI: 5.29-27.10%), respectively.

Although carboplatin was associated with the highest wastage percentage, gemcitabine and oxaliplatin contributed to the maximum financial losses of 45,100 INR (542.86 USD) and 37,606 INR (452.66 USD), respectively. Despite having a relatively lower wastage percentage of 9.69% (95% CI: 5.96-12.47%), paclitaxel resulted in the third-highest financial loss of 23,773 INR (286.15 USD). The cost of one vial of paclitaxel used in our setup was 2,647 INR (31.86 USD), whereas the cost of carboplatin was 1,700 INR (20.46 USD). These findings were consistent with a previous study by D'Souza et al., which reported that although paclitaxel wastage contributed to only 2.5%, the financial loss amounted to 10,910 INR (131.32 USD), which was the fourth highest [8].

One of the major factors that contribute to drug wastage is the availability of different vial sizes. In our hospital formulary, carboplatin and gemcitabine are available in only one vial size of 450 mg and 1,000 mg, respectively. This resulted in no matching of the commonest prescribed doses of 300 mg and 1,200 mg, respectively. In contrast, a study by Gopisankar et al. showed that gemcitabine had less wastage as it was available in multiple vial sizes (200 mg, 1,000 mg, 1,400 mg), whereas pemetrexed and doxorubicin, which were available only as single vials, accounted for the maximum wastage [7]. In our study, doxorubicin accounted for 8.87% wastage only, as it was available in two vial sizes of 10 mg and 50 mg. Hence, when the dosing is based on body weight/BSA, a certain degree of waste is inevitable.

To curb wastage, the hospital pharmacy can plan inventory as per the patient's requirements, which could be facilitated by making multiple vial sizes available for gemcitabine. Pharmaceutical companies should also be encouraged to manufacture multiple denomination vials of these drugs so that an effective drug dose can be arrived at with minimum wastage. Additionally, optimization of vial size can be achieved by considering the gender split, differences between patients in different geographies, and different indications. In scenarios where the larger vial size is perfectly divisible by the smaller vial size, such as 50 mg and 100 mg, wastage is markedly higher, and, ideally, such scenarios should be avoided [9].

In our setup, measures to decrease drug wastage were employed in 23 cases, which account for 14.6% of the total drug administrations. Of these, vial sharing and rounding of the dose were undertaken in 6.36% and 8.28% of drug administrations, respectively. However, the reasons for sparing use of these mitigation strategies at our setup are unclear and need further evaluation.

The Hematology/Oncology Pharmacy Association (HOPA) recommends that monoclonal antibodies and cytotoxic agents be rounded to the nearest vial size within 10% of the prescribed dose. According to the association, dose rounding within 10% in palliative care and within 5% in curative therapy can be implemented, based on the inference that it will not impact clinical safety or effectiveness [10].

The study by Dooley et al. demonstrated that cost savings of 4-14% can be achieved by dose rounding for drug administrations of doxorubicin, docetaxel, oxaliplatin, and gemcitabine [2].

A study on drug waste minimization in the oncology department conducted in Italy was able to reduce drug waste expenditure by 45% through the use of cost containment measures such as per pathology per drug distribution of chemotherapy sessions, the use of multi-dose vials, dose rounding, and appropriate vial size selection [11]. In a large setup like ours, it could be practically feasible to batch patients receiving common chemotherapy drug regimens for specific cancers. For instance, carcinomas of the stomach, colon, and rectum share the same drug regime (FOLFOX [folinic acid, fluorouracil, and oxaliplatin]), whereas carcinoma of the gall bladder and pancreas are treated with gemcitabine and cisplatin, and patients with breast and ovarian carcinoma receive carboplatin and paclitaxel administrations. Batching of patients scheduled to receive these drugs could make use of vial-sharing practices to reduce wastage.

Furthermore, all the drugs available at our pharmacy were single-dose vials. These vials must be either administered or discarded promptly once opened, and because a patient's body size is unlikely to match the amount of drug included in the vial, there is nearly always some drug leftover. The U.S. Pharmacopeia Convention 2008 Chapter <797> permits the sharing of drugs from a single vial, but only if it is used within six hours and stored appropriately [12].

While vial sharing can be an effective measure to reduce drug wastage, it can also pose a risk of microbial contamination if not handled properly. A study by Pritchard et al. demonstrated the potential for extending the beyond-use date for antineoplastic single-dose vials up to 28 days, provided they were stored in an International Organization for Standardization (ISO) Class 5 biological safety cabinet or refrigerator after puncture [13].

The use of a CSTD (Close System Transfer Device) is recommended to maintain sterility after opening the vial. PhaSeal® CSTDs have been proven to reduce exposure to hazardous agents during preparation and administration and are recommended by the United States Pharmacopeia, American Society of Health-System Pharmacists, and National Institute for Occupational Safety and Health as a safety measure to prevent occupational exposure to hazardous drugs [14].

Drug vial optimization (DVO) is another method used to minimize chemotherapy drug waste. It allows single-use vials to be used for multiple patients and has proven effective in using up chemotherapy injection waste from previous patients [15]. DVO can save on the cost of cytotoxic and chemotherapy agents, but the cost-effectiveness of these approaches needs to be evaluated in our setup.

One of the limitations of our study is that some cancers were not adequately represented in our study population due to institution-specific protocols. For example, head-neck face cancers and oral cavity cancers were not seen, probably because our institute lacks a radio-oncology unit. As a result, the wastage associated with the drugs used in those conditions could not be estimated. Additionally, as ours is a public sector hospital, monoclonal antibodies and small molecule therapies were not included in our hospital formulary, even though they are widely used in the private sector. Therefore, the cost associated with their wastage needs to be evaluated in future studies.

## Conclusions

Minimizing drug wastage in cancer care requires a comprehensive approach that includes appropriate vial sizes, dose rounding, and vial sharing. Pharmaceutical companies must consider drug vial size optimization to reduce wastage. By implementing these strategies, institutions can significantly reduce drug wastage and improve the efficiency of cancer care, and it will also help to reduce the economic losses that are very important for developing countries like India.
